# Disruption to Transformation: Engaged Research

**DOI:** 10.1093/geroni/igab046.573

**Published:** 2021-12-17

**Authors:** Carol Geary, Katherine Abbott, Erin McGaffigan

## Abstract

With changes in funders’ requirements, engagement of persons with “lived experience” in the planning, conduct, and dissemination of research is increasingly common. Although patient and stakeholder engagement is expected, the body of literature describing necessary structures and processes is severely limited. Therefore, the purpose of this symposium is to aid in the movement of engaged research from disruption to translation. To do so, we will describe gaps in researcher knowledge and skills associated with patient and stakeholder engagement; structures and processes in current use with older adults, and outcomes associated with engagement. We will begin by sharing findings within advisory board models of engagement. Dr. Lessem will describe the Sage Resource Project researcher needs assessment. Researchers (N=103) shared both their engagement interests and their perceived knowledge and capability gaps. Dr. Berman will describe training developed within the same project to overcome researchers’ perceived gaps. Then, Dr. Roes will describe a qualitative evaluation of persons with dementia perspectives on rewards and benefits of advisory board involvement. Our final two presenters will describe engagement using a variety of engagement approaches. Dr. Douglas will share experiences with adapting Montessori philosophies and processes to engage CNAs in development of innovative programming for dementia care within a long term care environment. Finally, Dr. Geary will share her team’s findings from interviews completed using appreciative inquiry with sites with over five-years’ experience engaging patients in research. Each site has developed unique infrastructures and processes to most effectively achieve desired outcomes.

